# Spectrum of Spondyloarthritis Among Chinese Populations

**DOI:** 10.1007/s11926-022-01079-1

**Published:** 2022-07-13

**Authors:** Shangzhu Zhang, Linyi Peng, Qingyang Li, Jinwei Zhao, Dong Xu, Jiuliang Zhao, Qian Wang, Mengtao Li, Wen Zhang, Xinping Tian, Jinmei Su, Xiaofeng Zeng

**Affiliations:** 1 Department of Rheumatology and Clinical Immunology, Peking Union Medical College Hospital, Chinese Academy of Medical Sciences & Peking Union Medical College; National Clinical Research Center for Dermatologic and Immunologic Diseases (NCRC-DID), Ministry of Science & Technology; State Key Laboratory of Complex Severe and Rare Diseases, Peking Union Medical College Hospital (PUMCH); Key Laboratory of Rheumatology and Clinical Immunology, Ministry of Education,, No.1 Shuaifuyuan, Dongcheng district 100730 Beijing, China; 2 National Clinical Research Center for Dermatologic and Immunologic Diseases (NCRC-DID), Ministry of Science & Technology, 300191 Tianjin, China

**Keywords:** Spondyloarthritis, Chinese, HLA-B27, Ankylosing spondylitis, Epidemiology, Treatment, Juvenile idiopathic arthritis, Psoriatic arthritis, Enteropathic SpA

## Abstract

**Purpose of Review:**

This review aims to emphasize interesting and important new findings with a focus on the spectrum of spondyloarthritis (SpA) in China.

**Recent Findings:**

Over the past decade, significant advances have been made in the investigation of SpA epidemiology, the exploration of genetic and environmental risk factors, the identification of clinical features, and the updating of treatment protocols in the Chinese population. The prevalence of ankylosing spondylitis (AS) in China is 0.20–0.42%, and the prevalence of HLA-B27 in AS patients is 88.8–89.4%. HLA-B*2704 is the most common subtype in Chinese AS patients, followed by HLA-B*2705. HLA-A*01, more precisely HLA-A*01:01, may be associated with psoriatic arthritis (PsA). Tumor necrosis factor inhibitors and IL-17A inhibitors have been shown to be effective and safe for AS patients in China. Juvenile-onset AS is relatively rare, accounting for only 9.1% of the AS population. The prevalence of arthritis related to inflammatory bowel disease is 6.9 to 7.2%. A Chinese study showed that the most frequently prescribed medication was methotrexate (66.4%). Biological agents were prescribed in only16.4% of patients with PsA.

**Summary:**

This review summarizes the latest research in the epidemiology, pathogenesis, clinical manifestations, and management of SpA among Chinese populations. Multiple HLA associations with SpA have also been described, and it is hoped that discoveries of such ethnic-specific risk factor(s) and understanding of their pathological mechanisms may potentially lead to newer targeted therapies for the Chinese populations worldwide.

## Introduction

Spondyloarthritis (SpA) is a group of diverse interrelated inflammatory arthritides with multiple clinical features and common genetic predisposition, histopathological and likely etiopathogenic features [1]. The term SpA includes ankylosing spondylitis (AS), a subgroup of juvenile idiopathic arthritis (JIA), psoriatic arthritis (PsA), arthritis related to inflammatory bowel disease (IBD), and reactive arthritis (ReA). AS is considered to be the most typical subtype (the prototype) of SpA. Various clinical forms include axial features, peripheral arthritis, enthesitis, tendon lesions, and extra-articular features, such as uveitis, psoriasis, and inflammatory bowel disease [2]. The epidemiological data for these closely related diseases vary among ethnic groups and regions [3].

China is the world’s most populous country, with a population of more than 1.4 billion, accounting for approximately 1/5th of the world’s population. Approximately 40–45 million people of Chinese ethnicity currently live outside their ancestral homeland, and eventually established foundations for substantial communities in the Asia, Americas, Australia, the Pacific and Indian Ocean islands, Europe, and parts of Africa [4, 5]. With such a large population base and distribution globally, it is of great significance to understand the spectrum of SpA among the Chinese populations.

With the continuous updating of SpA classification criteria, the development of diagnostic methods including MRI, the gradual exploration of pathogenesis, and the upgrading of therapeutic methods such as TNF-inhibitors (TNFis) and IL-17A inhibitors therapy, an increasing number of studies have been widely carried out in the Chinese population and are worthy of attention [1, 2, 6–8]. Given the limited literature on ReA reported for the Chinese population, SpA in this review includes AS, JoAS, PsA, and arthritis related to IBD. This review will focus on the latest research and views on the epidemiology, pathogenesis, clinical manifestations, and management of SpA among Chinese populations.

## AS

### Epidemiology

#### Incidence

A large, community-based investigation conducted in Kailuan in 2006–2015 (*n* = 129.681, follow-up over 10 years) showed an AS incidence rate of 5.32/100,000 person-years, decreasing with age, and the incidence of AS was 3.16 times higher in men than in women (6.35/100,000 person-years vs. 2.01/100,000 person-years) [9••]. Among AS patients, 45.0% were farmers, 32.5% were workers, and 11.4% were freelancers [10].

In European and American countries, such as Denmark, Norway, Canada, and America, the incidence of AS is 1.74/100,000 person-years, 7.26/100,000 person-years, 15.00/100,000 person-years, and 3.1/100,000 person-years [11–14], respectively. The global incidence of AS fluctuates over a wide range, in which the Han population in northern China is at a lower-middle level. However, it is worth noting that China has 56 ethnic groups, and at present, there is a lack of multicenter multiethnic epidemiological surveys to capture the whole picture.

#### Prevalence

Reviewing the epidemiological survey of AS conducted in China in the past 15 years, three cross-sectional studies on military populations (*n* total = 54,474) showed that the prevalence rate of AS in the Chinese military population was 0.17–0.44% [15–17], while seven cross-sectional studies based on community populations (*n* total = 55,959) showed that the prevalence rate of AS in the community population was 0.20–0.42% (Table 1) [18–24]. A comparison of two non-overlapping meta-analyses from 2005 to 2019 and 1994 to 2006 showed that the prevalence of AS in China was higher in 2005 to 2019 than in 1994 to 2006 (0.29% vs. 0.24%) [25•, 26]. In comparison, the prevalence of AS ranges from 0.20 to 0.55% in the USA [27], 0.25% in Europe, 0.02% in Africa, 0.11% in the Middle East, 0.16% in East Asia, 0.07% in Southeast Asia, and 0.06% in South Asia [28].

**Table 1:** Data on the prevalence of ankylosing spondylitis in the Chinese population

Author	Publication year	Province/city	Research type	Diagnostic criteria of AS	Sample size	Male	Female	Mean prevalence of AS (%)	Sampling resource
Zeng et al. [18]	2008	Nanning, Guangxi Province	Cross-sectional study	New York criteria (1984)	14,233	7497	6736	0.21	Community
Chen et al. [19]	2005	Beijing	Cross-sectional study	New York criteria (1984)	1982	1025	957	0.3	Community
Dong et al. [20]	2006	Taiyuan, Shanxi Province	Cross-sectional study	New York criteria (1984)	3915	1858	2057	0.2	Community
Wang et al. [21]	2011	Zaozhuang, Shandong Province	Cross-sectional study	New York criteria (1984)	12,536	6395	6141	0.42	Community
Ye et al. [22]	2006	Shenzhen, Guangdong Province	Cross-sectional study	New York criteria (1984)	5922	2659	3263	0.37	Community
Liao et al. [23]	2009	Qingyuan, Guangdong Province	Cross-sectional study	New York criteria (1984)	13,315	7013	6302	0.22	Community
Zeng et al. [24]	2015	Shantou, Guangdong Province	Cross-sectional study	New York criteria (1984)	4056	1948	2108	0.3	Community
Wu et al. [15]	2008	Xinjiang Uygur Autonomous Region, Qinghai Province, Shandong Province, Gansu Province, Shanxi Province	Cross-sectional study	New York criteria (1984)	21,750	20,250	1500	0.21	Military
Wang et al. [16]	2013	Anhui, Sichuan, Shanxi, Shaanxi and Henan Provinces	Cross-sectional study	New York criteria (1984)	1164	1164	0	0.17	Military
Lin et al. [17]	2015	Fujian Province	Cross-sectional study	Chinese Society of Rheumatology criteria	31,560	29,120	2440	0.44	Military

### HLA-B27

The general prevalence of HLA-B27 markedly varies worldwide [29]. Prevalence of HLA-B27 in the Han population ranges between 3.6 and 5.7% [30], lower than the 8% reported in Europe [28]. Two large-scale multicenter studies conducted statistical analyses on the prevalence of HLA-B27 in Chinese patients with AS, showing that 88.8–89.4% of AS patients are HLA-B27 positive in China [31••, 32]. Among AS patients in Korea, Europe, Canada, and Latin America, the prevalence of HLA-B27 was 94.8%, 83%, 79%, and 71%, respectively [33–36].

There is marked polymorphism of HLA-B genes, including HLA-B27, and an updated naming system was developed to encompass these variants. There are more than 224 transcribed proteins (HLA-B27 subtypes or allotypes) that differ by one or more amino acid substitutions and show an extremely varied racial/ethnic prevalence throughout the world. HLA-B*27:05 is the most widely distributed and disease-associated subtype in the world [37]. But in the Chinese Han population, HLA-B*2704 is the most common subtype (55.0–88.0%) and that of HLA-B*2705 is 10.1–42.4% [38•, 39, 40•, 41]. Similarly, HLA-B*2704 is the most predominant subtype in AS patients in China, followed by HLA-B*2705. In contrast, HLA-B*2705 and HLA-B*2702 are the main subtypes in the Caucasian population; and HLA-B*2702 appears more frequently in the Jewish and Arab/Berber populations [42]. Table 2 lists the associations of HLA-B27 and its subtypes in AS, juvenile-onset AS (JoAS) in Chinese population.

**Table 2:** HLA-B27 associations with spondyloarthritis in Chinese population

SpA	HLA-B27 association					Other HLA-associations
	General	HLA-B*2704	HLA-B*2705	HLA-B*2702	HLA-B*2715	
AS	88.8–89.4% [31••, 32]	55.0–88.0% [38•, 39, 40•, 41]	10.1–42.4% [38•, 39, 40•, 41]	-	1.0% [40•]	HLA-DP/DQ, HLA-B [42–44]
JoAS	78.0–94.6% [45–48]	49–82.8% [49, 50]	7.59–45.5% [49, 50]	1.82% [50]	2.07% [49]	
PsA	15.6% [51]	-	-	-	-	HLA-Cw*12, HLA-A*01:01, HLA-A*01 [52, 53•]

### Environmental Factors

The etiology of AS remains unclear, yet in addition to genetic risk factors, including HLA-B27, environmental factors, such as geographical location, lifestyle, dysbiosis of intestinal microorganisms, infection, and physical exercise, have also been implicated in AS pathogenesis. A two-center cross-sectional study (*n* = 807) compared the clinical characteristics of AS patients in northern and southern China based on their native places and found that the proportion of patients with peripheral joint onset was significantly greater in the northern group than in the southern group, while the proportion of patients with axial joint onset showed the opposite result [54].

As reported, the Bath Ankylosing Spondylitis Disease Activity Index (BASDAI) and Bath Ankylosing Spondylitis Functional Index (BASFI) were significantly higher in smokers than in nonsmokers [55, 56]. AS patients who smoke have a longer course of disease, a high disease activity index, a poor disease function index, higher inflammatory indicators, and increased inflammatory manifestations on MRI [55].

A study that analyzed the feces of untreated AS patients (*n* = 85) showed increased oxidative phosphorylation, lipopolysaccharide biosynthesis, and degradation of glycosaminoglycans. Common characteristics associated with autoimmunity were also identified in AS intestinal microorganisms, such as *Bacteroides fragilis* and type III secretion systems (T3Ss), suggesting that the gut microbiota of untreated AS patients is disturbed [57].

A large community-based cohort study (*n* = 121,656) showed that baseline hs-CRP was positively associated with the risk of future AS and suggested that CRP plasma concentrations may predict the risk of future AS in the Chinese population [9••]. Using the same cohort, it was also found that adherence to ideal physical activity (> 80 min/week) may reduce the risk of developing AS [58].

### Clinical Manifestations

The mean age of onset in AS patients in China was 29.2 years [32]. Zhang et al. [31••] showed that the age of AS onset in HLA-B27 carriers was lower than that in non-HLA-B27 carriers (26.2 ± 9.45 vs. 31.3 ± 12.3), and the disease duration (5 years vs. 3 years) and the prevalence of a family history (24.3% vs. 11.5%) were higher in HLA-B27 carriers than in non-HLA-B27 carriers. Similar results have previously been reported in studies of populations of European descent [59].

With regard to extra-articular manifestations, patients with HLA-B27 had a lower prevalence of psoriasis (0.7% vs. 2.5%) and a higher prevalence of uveitis (11.2% vs. 5.1%) [31••, 32]. HLA-B27 typing revealed that the incidence of uveitis was associated with the subtypes HLA-B*2704 and HLA-B*2705 [38•, 39, 42]. In addition, patients with HLA-B27 expressed higher disease activity as measured by C-reactive protein (CRP) and ASDAS-CRP [31••].

#### Skeletal Manifestations

As in other countries, the most common clinical manifestation of AS in patients in China is lower back pain and stiffness [60]. Lou et al. [61] found that 68.1% (258 of 379) of the patients had morning stiffness of the low back, 87.1% had stiffness, 97.1% had pain, 100% had limited activity, and 39.31% had deformity. Approximately 28.8–61.1% of patients had low back pain as the first manifestation [10, 61].

In a cohort study (*n* = 449), patients with a longer disease duration were more likely to show thoracic spine involvement at the early stage of disease onset. AS patients with a disease duration of ≥ 5 years were prone to thoracic spine involvement, AS patients with a disease duration of ≥ 10 years were prone to anterior chest wall pain, and the proportion of patients with limited thoracic mobility increased with a disease duration of ≥ 5 years [62].

The hip joint is frequently involved in joint injuries in Chinese patients with AS and is the most critical site of disability in 2 [63]. Approximately 59.0–73.7% of patients in China have hip joint involvement [64, 65]. Li et al.’s [64] study (*n* = 331) found that the age of AS onset in the hip involvement group was lower than that in the non-hip joint involvement group, and the level of CRP was higher than that in the non-hip involvement group, suggesting that younger age may be a risk factor for AS with hip involvement. Han et al.’s [65] study showed that a long disease duration, high state of disease activity, young age of onset, low Harris score of hip joint function, and short walking distance were independently associated with the degree of radiological progression in patients with AS hip lesions. In terms of manifestations of AS, compared to patients without HLA-B27, those with HLA-B27 had a significantly higher likelihood of hip joint involvement (33.2% vs. 25.7%) but a lower likelihood of peripheral arthritis (27.0% vs. 36.2%) and dactylitis (6.3% vs. 9.7%) at enrollment [31••].

According to the Chinese Spondyloarthritis Registry, 33.9% of AS (*n* = 4875) patients currently or previously had peripheral arthritis, 29.7% of patients had current peripheral arthritis swollen, 66.2% had enthesitis, and 22.7% had current or past heel pain [66•].

In various countries, the clinical characteristics of AS patients are similar. However, the prevalence of peripheral arthritis in AS patients in China was lower than that in AS patients in Korea (47.1%) and India (65.7%) [33, 67].

#### Eye Disease

Uveitis is the most common extra-articular manifestation of AS [68]. The prevalence of acute anterior uveitis (AAU) in Chinese AS patients ranges from 9.7 to 16.2% [66•, 69–71], which is lower than the 23% in the USA and the 25.8–32.7% in Europe [72–74]. In Chinese patients, the manifestation of uveitis has also been confirmed by a number of studies to be closely related to disease duration and other manifestations of AS. Three studies jointly showed that the average disease duration in the uveitis group was longer than that in the non-uveitis group (7.84 ± 7.45 years vs. 4.14 ± 4.43 years), and the proportion of peripheral joint involvement in the uveitis group (51.4–77.8%) was higher than that in the non-uveitis group (32.5–47.6%) [70, 71, 75].

### AS in Males and Females

Several studies have shed light on the differences in clinical manifestations of AS between males and females in China. In mainland China, the male:female ratio of AS prevalence is 2.8:1 (0.42%:0.15%) [25•]. However, the global male:female ratio of AS prevalence is 2.58:1 (0.31%:0.12%) [28], which is a little lower than that in mainland China.

A multicenter cross-sectional large survey including across China found that males were significantly younger than females at the time of onset and diagnosis [32]. The prevalence of HLA-B27 and JoAS was also significantly higher in males than in females [32, 45, 47, 76], and the incidence of AAU was slightly higher in males (11.3% vs. 7.4%), with higher C-reactive protein levels and a higher ESR in males [32]. In addition, male SpA patients had more HBV infection than females (26.2% vs. 8.3%) and were more likely to develop osteoporosis (30.5% vs. 14.3%) [77].

### Management

#### Physiotherapy

Exercise therapy is an important intervention for enhancing function and improving quality of life in AS patients, and positive effects have been observed in AS patients in Europe [78]. Sun et al.’s [79] study demonstrated the efficacy of ultrasound and exercise therapy in Chinese AS patients.

#### Pharmacotherapy

NSAIDs are the cornerstone of pharmacological intervention for AS in China, but there have been few noteworthy trials in recent years.

As reported, TNFis can significantly reduce the signs and symptoms of Chinese patients with active AS and improve their body function and quality of life. Etanercept, adalimumab, and golimumab have been shown to achieve better ASAS20 response rates than placebo [80–82]. In addition, a meta-analysis showed that compared with the Chinese population, the Caucasian population had higher ASAS40 response and partial remission, but the incidence of adverse events was relatively lower in the Chinese population [83]. The efficacy and safety of HS016, a biosimilar candidate for adalimumab, were also found to be similar to adalimumab in a phase III clinical trial [84]. However, it is worth noting that the current guidelines for the treatment of AS [85] indicate that TNFi therapy needs to be used for a long period of time, and cases of relapse and infections have been observed, particularly in Asian patients, after completion of short-term TNFi treatments [3, 86, 87]. A prospective cohort study found that patients with complete TNFi withdrawal (89.0%, *n* = 57) had a considerably greater recurrence rate than patients with TNFi reduction (30.8%, *n* = 26) and patients with partial TNFi withdrawal (27.3%, *n* = 22) [88].

Secukinumab, an IL-17A antibody, was approved by the FDA in 2015 for the treatment of AS and has been shown to safely improve signs and symptoms, physiological function, and disease-specific quality of life in active Chinese patients with AS [89, 90].

It is worth mentioning that approximately 38% of patients worldwide use biological disease-modifying antirheumatic drugs (bDMARDs). According to data from 22 countries, the average usage rate of bDMARDs varies from 5% in China to 74% in Belgium [91]. China has the lowest bDMARD utilization rate. In many countries and regions (including China), economic constraints and/or a lack of drug resources may be important factors that limit the clinical use of bDMARDs and cause a considerable number of AS patients to accept conventional synthetic disease-modifying antirheumatic drugs (csDMARDs) at a much lower price [92].

CsDMARDs, including methotrexate, sulfasalazine, hydroxychloroquine, and thalidomide, are typically used as second-line drugs for AS treatment; csDMARDs are usually ineffective for axial manifestations of SpA but effective in specific cases of peripheral AS [84]. An observational study (*n* = 320) showed that sulfasalazine and thalidomide are the most commonly used csDMARDs in China, with a cumulative use of 8.9 ± 4.1 months and 9.1 ± 4.7 months, respectively [92]. The addition of csDMARDs could reduce the disease activity of AS in the Chinese population compared with NSAID monotherapy [92]. Qiu et al. [93] found that the new immunosuppressant leflunomide (LEF) could significantly reduce the BASDAI, peripheral joint tenderness, and swollen joint count in Chinese AS patients, and improve the ASAS20 response, with similar efficacy to sulfasalazine and a lower incidence of adverse reactions (18.3% vs. 31.7%).

## Juvenile Onset AS

### Prevalence

A multicenter, cross-sectional survey conducted in five cities in China from September 2010 to April 2014 (*n* = 1251) showed that JoAS constitutes 9.1% of AS patients [32], at the lower end of the 9 to 21% range reported in the Caucasian population [94, 95]. However, single-center cohort studies in China [45, 49, 96, 97] noted higher proportion of JoAS at 20–36%, possibly due to selection bias caused by the use of different patient enrollment strategies somewhat determined by the varying aims of these studies.

### HLA-B27

The prevalence of HLA-B27 in JoAS patients is 78.0–94.6%, similar to those observed in adult-onset AS (AoAS) [45–48].

HLA-B27 subtype distribution among the JoAS patients was found to be dissimilar in two studies, one involving 145, and the other 55 patients [49, 50]. The prevalence of HLA-B27*04 and HLA-B27*05 in these studies was 82.8% versus 49% and 7.59% versus 45.5%, respectively.

### Clinical Manifestations

The average age of onset in Chinese patients with JoAS has been reported to be around 13.8 years [32, 97]. The prevalence of a family history of AS ranges from 16.0 to 28.6% and is similar to that among AoAS patients [32, 45, 97].

Mou et al. [49] reported that the prevalence of enthesitis was higher in JoAS patients (*n* = 145) than in AoAS patients (*n* = 310) (63.5% vs. 50.0%). A study showed that 97.9% of JoAS patients (*n* = 47) had enthesitis with 58.7% of the patients having enthesitis as the initial symptom [96]. However, the prevalence of enthesitis as the initial symptom was only 13.4% in another study (*n* = 67) [48]. Liu et al. [97] reported higher prevalence of peripheral arthritis in JoAS patients (*n* = 75) relative to that in AoAS patients (*n* = 275) (45.3% vs. 18.9%). Peripheral arthritis was the initial symptom in 37–72% of the JoAS patients [45, 48, 96, 98]. Hip joint involvement affecting 26.7–61.7% of the patients with JoAS was also observed more frequently than in AoAS patients [49, 96, 97]. Surgical treatment (primarily hip-joint replacement) was performed nearly six times more often than AoAS patients (4.90% vs. 0.76%) [32].

The prevalence of acute uveitis in JoAS patients (10.2–36.2%) was found to be similar to that of the AoAS group in most of the studies [32, 48, 96], except for the study by Liu et al. [97] which reported a higher prevalence among JoAS patients (17.3% vs. 6.6%). Low bone mineral density (BMD) was detected in 16.1% of patients with JoAS. BMD of lumbar spine was negatively correlated with BASDAI, BASFI, spine pain, ESR, and CRP [99].

### Delay in Diagnosis

A multicenter cross-sectional large survey in China found that the median time of delay in diagnosis was 2 years in JoAS patients (*n* = 105) which was similar to that in AoAS patients (*n* = 1049) [32]. Lin et al. [96] reported the mean time of delay in diagnosis was similar in JoAS patients (*n* = 47) and AoAS patients (*n* = 122) (5.7 ± 6.3 years vs. 4.6 ± 6.9 years). But another study reported longer time of delay in diagnosis in JoAS patients (*n* = 50) than in AoAS patients (*n* = 89) (5.32 ± 5.26 years vs. 2.85 ± 3.82 years) [45].

## Psoriatic Arthritis

Psoriatic arthritis (PsA) is a chronic systemic inflammatory disease that involves both peripheral joints and the axial skeleton and is associated with psoriasis [100]. Psoriasis patients with HLA-B*27 and/or -Cw*12 may have a higher risk of developing PsA [52]. Chen et al. [53•] found that HLA-A*01:01 and HLA-A*01 may be susceptibility genes associated with PsA and confirmed the association of four loci with PsA in a Chinese Han population. From population-based surveys in China, the prevalence of PsA in China appeared to be similar to that in the rest of the world, ranging from 10 to 100 per 100,000 population [30].

A recent study that enrolled three hundred Chinese PsA patients showed that 15.6% of patients were HLA-B27 positive, and 37.8% patients reported a family history of psoriasis or PsA. Psoriasis presented earlier than arthritis in 74.0% of patients, while 16.6% of patients presented with arthritis before psoriasis. The polyarticular type was most common, with proximal interphalangeal joints as the most frequently involved joints. Axial skeleton involvement was found in 15.4% of patients. Dactylitis was observed in 31.3% patients [101••]. Other studies showed that the prevalence of dactylitis and enthesitis ranged from 15.4 to 71.4% and from 7.8 to 59.1%, respectively [102]. Nail lesions were common in PsA, affecting 30.2–97.0% of the patients, and were significantly more prevalent than in psoriasis patients without arthritis in one study [102]. Eye lesions were rarely reported in PsA patients in Asia (1.7–3.9%) [102]. A study showed that PsA-associated fatigue is prevalent and is associated with disease activity, impact, and chronicity [103•]. Another study showed that patients with PsA had a higher Psoriasis Area and Severity Index score and a higher prevalence of the erythrodermic type than psoriatic patients without arthritis [30, 104]. A study with 97 patients who completed a follow-up survey showed that Chinese patients with PsA had poor physical function and quality of life. One-fifth of the patients experienced a deterioration of physical function over time. Joint damage and baseline physical function were important factors associated with a deterioration of physical function over time in patients with PsA [105].

The Classification of Psoriatic Arthritis (CASPAR) criteria performed well in a Chinese population, which is very different from the populations for which they were developed. The sensitivity and specificity of CASPAR criteria when applied to a Chinese population were calculated to be 98.2% and 99.5%, respectively, similar to the reported values in European populations [106].

A study demonstrated that methotrexate was more effective, with fewer adverse effects, in patients with psoriasis who did not have PsA than in patients who presented with both psoriasis and PsA [107•]. A recent Chinese study showed that the most frequently prescribed medication was methotrexate (66.4%). Biological agents were prescribed in 16.4% of patients [101••]. In Chinese patients with psoriasis and concomitant PsA, secukinumab demonstrated high efficacy outcomes at week 12 which were sustained through week 52 [108•].

## SpA with Inflammatory Bowel Disease (IBD)

In the Chinese AS Prospective Imaging Cohort (CASPIC), the prevalence of IBD in AS patients was 6.9–7.2% [109, 110••], slightly higher than the prevalence of 3.7–4.5% in Europe [111, 112].

Based on Taiwan National Health Insurance Research Database (NHIRD) from 2005 to 2012, the overall incidence of IBD was lower in the AS group than in the non-AS group, but without reaching statistical significance (1.41 vs. 1.79 per 1000 person-years, incidence rate 0.79, 95% CI 0.48–1.28; *p* = 0.332) [113]. Interestingly, another retrospective cohort study based on the same database from 2000 to 2012 showed that patients with AS had a higher risk of IBD than the non-AS group in the subgroup aged < 40 years (HR: 2.85, 95% CI: 1.51–5.40) [114].

In a single-center retrospective study, AS patients with IBD (*n* = 64) were found to be older and to have a longer disease duration than those without IBD (*n* = 829). Furthermore, AS patients with IBD had more frequent involvement of the cervical spine and thoracic spine, higher disease activity, poorer functional and general health indices, and an increased chance of developing psoriasis and uveitis [110••].

## Conclusion

Compared with other countries in the world, AS in China has some unique epidemiological characteristics, including a relatively high disease prevalence. Over the past 20 years, the effectiveness of bDMARDs, such as TNFis and IL-17A inhibitors in the Chinese population, has been consistently shown. But their high prices decrease their general affordability and create a major obstacle to their widespread clinical use in China. Thus, a considerable number of AS patients in China can access only csDMARDs at a much lower price, but with no clear evidence of efficacy. Similar to AS, JoAS is closely associated with HLA-B27. Susceptibility genes for PsA in Chinese population include HLA-Cw*12, HLA-A*01:01, and HLA-A*01 in addition to HLA-B27.

Through research in the past few decades, multiple HLA associations with SpA have been found in the Chinese population, and it is hoped that discoveries of such ethnic-specific risk factor(s) and understanding of their pathological mechanisms may potentially lead to newer targeted therapies for the Chinese populations worldwide.
